# Using the Constitutionality Framework to Understand Alliances, Collective Action, and Divisions Between Indigenous and Peasant Communities in the Chaco Salteño

**DOI:** 10.1007/s10745-022-00337-1

**Published:** 2022-08-12

**Authors:** Maurice Tschopp, Carla Inguaggiato, Rodrigo Chavez Saravia, Michele Graziano Ceddia

**Affiliations:** 1 Centre for Development and Environment, University of Bern, Mittelstrasse 43, CH3012 Bern, Switzerland; 2 National University of Salta (UNSa), Salta, Argentina

**Keywords:** Constitutionality, Collective action, Common-pool resources, Dry Chaco, Deforestation

## Abstract

This article analyzes bottom-up institution-building processes in a region considered deforestation and environmental degradation hotspot. Utilizing the constitutionality approach developed by Haller, Acciaioli, and Rist (2016), we examine two recent cases of bottom-up institution-building in the department of Rivadavia, Chaco Salteño, Argentina. We highlight the similarities and differences between both constitutionality processes and identify various weaknesses in the two cases. We argue that constitutionality, understood as a process, has occurred to different (incomplete) degrees in each case. Finally, we show that external catalyzing agents play a decisive role in enabling or hampering the constitutionality process. Our study contributes to the literature on common-pool resource governance by highlighting how collective action can lead to participatory-development processes.

## Introduction

The Dry Chaco in South America is the second-largest forest ecosystem. It is also one of the world’s deforestation hotspots. Deforestation in the Chaco has been driven primarily by cropland and pasture expansion, especially for soy production and cattle ranching (Baumann et al., 2017; Fehlenberg et al., 2017; Piquer-Rodríguez et al., 2018; Waroux et al., 2018). The corresponding massive conversions of land have dramatic consequences in terms of losses of ecosystem services (Baldassini and Paruelo 2020; Volante et al., 2012), which primarily impact forest users such as small-scale farmers and indigenous people. The Dry Chaco is home to over 40 indigenous peoples, who speak 29 languages (Schaumberg, 2020), and small-scale cattle ranchers, also called *criollos.* These groups have been subjected to evictions by large-scale landowners and are perceived as marginalized in the policy arena because they rely on extensive silvopastoral production systems and lack land tenure security (Naharro, Alvarez, and Flores Klarik 2010).

The current process of agricultural expansion in the Chaco has been referred to as a process of “accumulation by dispossession” by many observers (Zarrilli, 2020; Cáceres, 2015; Gorenstein and Ortiz 2016; Lende 2015), highlighting how social-environmental conflicts between the agro-industrial sector and peasant and indigenous communities lies at the center of agricultural expansion. Some scholars have emphasized the ambiguous role of the Argentinian state in this process (Biocca, 2015). On the one hand, the state created legal and political conditions facilitating extensive agricultural expansion. It often remained passive in front of evictions of forest users and human rights violations – a passivity described as “tolerated illegalism of rights” by Gebara (2018). On the other hand, the state provided support for the land claims of indigenous and peasant communities and enacted legislation aiming to enhance the legal protections of these actors (Biocca 2017).

Overall, while several policies have sought to address the vulnerability of local forest users, and several legal instruments could eventually provide land tenure security to these marginalized groups, there remains a gap between the rights granted by Argentina’s constitution and the actual existing, locally enforced laws on land tenure and indigenous rights. In a recent review of the implementation of land tenure legislation in three South American countries, Gebara (2018) indicated that local leadership and activism by indigenous groups were essential factors contributing to legal recognition and land tenure regularization. In Salta province, several researchers have highlighted that protests and local actors’ participation helped reinforce local institutions (Hufty, 2008; Kolesas, 1998). Other studies have focused on conserving and managing social and natural capital in a context characterized by increasing pressure from the agro-industrial sector (Auer et al., 2020).

However, there is a lack of understanding of the specific conditions under which local activism can succeed in helping to strengthen local land rights in the region. The legal system defines special rights for indigenous people, on the one hand, and criollo smallholders, on the other (Brent, 2015). Nevertheless, while the two groups may be embedded in different legal systems and have very distinct livelihoods, they often face similar struggles in obtaining formal recognition for their land access and the cultural and social norms that shape their traditional forms of land use. The question arises: Why do some land regularization processes succeed while others fail?

The paper analyzes specific conflicts and collaboration occurring between *criollos* and indigenous people in Argentina’s Dry Chaco by applying the constitutionality approach developed by Haller, Acciaioli, and Rist (2016). This approach provides a framework describing the conditions for bottom-up institution-building by local actors and analyses how these institutions can contribute to the sustainable use of natural resources. The constitutionality approach recognizes institutions as outcomes of negotiation processes between different actors. As such, the resulting institution has to be regarded as a compromise reflecting different levels of bargaining power (Chabwela and Haller 2010; Haller, Acciaioli, and Rist 2016). The constitutionality approach addresses a “blind spot” in natural resource governance. It conceptualizes how institutions can emerge from conflicts over resources management and how these institutions can provide collective benefits for all actors, even in situations characterized by a high level of power asymmetries.

This approach identifies six conditions associated with successful institution-building, namely: (1) Emic perceptions of factors creating the need for new institutions; (2) participatory processes addressing power asymmetries; (3) preexisting institutions; (4) outside catalyzing agents (fair platform); (5) recognition of local knowledge; and (6) higher-level acknowledgment of the new institutions. In contrast to Ostrom’s institutional analysis and development framework (Ostrom, Gardner & Walker 1994), the constitutionality approach addresses local actors’ participation, agency, and power behind the institution-building process (Haller, Acciaioli, and Rist 2016). The original framework builds on four different case studies from Zambia, Mali, Indonesia, and Bolivia. Since then, various scholars have applied it to new cases, and the framework has been expanded to other contexts. Belsky and Barton have used it to analyze the development of a Blackfoot Community conservation area in Montana (Belsky and Barton 2018). Eid and Haller have applied it in connection with a Druze Arab minority in Mount Carmel (Eid and Haller 2018). Gambon has examined the development and limits of the constitutionality process regarding establishing a nature reserve in Bolivia (Gambon and Rist 2018). Faye et al. have examined the creation of new institutions in Senegal (Faye, Haller, and Ribot 2018). Finally, Ochoa Garcia has applied the framework to water governance and environmental justice in Mexico (Ochoa-García and Rist 2018). Most of these studies describe *successful* cases of bottom-up institution-building, although they sometimes identify some weaknesses associated with the constitutionality process itself. For instance, Gambon and Rist (2018) suggest that creating the pillon-lajas reserve in low-land Bolivia resulted in higher institutional fragmentation and ultimately an unsatisfactory compromise due to ontological differences.

In the following, we examine two specific cases of bottom-up institution-building: (1) the creation of Mesa de Gestión in Santa Victoria Este; and (2) the process of titling state land on behalf of indigenous and peasant communities in Rivadavia Banda Norte. We highlight the similarities and differences between both constitutionality processes and identify several issues that have slowed down or halted constitutionality in these cases. The text is structured as follows: Section two presents our region of study (the department of Rivadavia, in the province of Salta) as well as its history; section three describes our methods; section four analyses the two cases of constitutionality; and section five discusses our results, focusing on key weaknesses of the constitutionality process and emphasizing the role of external actors and particular barriers. We conclude by discussing the policy implications of our research.

## Region of Study: Historical Conflicts and the Legal Response to Deforestation

Our research focuses on two municipalities in the province of Salta in northern Argentina. The Province of Salta has experienced some of the country’s highest deforestation and environmental degradation rates in the last two decades (Alcañiz & Gutierrez, 2019; 2020; Vallejos et al., 2021). Large farms, which make up less than 10% of all agricultural units, are present in more than two-thirds of the areas where conflicts among land users are occurring (Seghezzo et al., 2017)^1^.

### Historical Background

While the Dry Chaco is currently considered one of the most critical agricultural frontiers in South America, its climate was initially deemed too harsh for cultivation by Spanish colonizers. Many areas in the territory were left virtually untouched during the first phases of the colonization. This changed in the 19th century based on the Argentinian state’s territorial expansion and corresponding military campaigns. The Argentinian army carried out several military campaigns against indigenous communities, such as the one led by Benjamin Victoria to secure control of the Bermejo River. These campaigns killed thousands of indigenous people and strongly affected subsequent indigenous resistance movements in the Chaco region (Gordillo and Hirsch 2003).

Nevertheless, several villages and settlements in Argentina’s Dry Chaco still bear the names of military commanders that led campaigns in the area or otherwise indicate the military past of settlements (Coronel Juan Sola, Fortin Dragones). Notably, these military campaigns occurred parallel to efforts by the provinces of Salta and Jujuy to repress indigenous uprisings in the highlands (Bernal, 1984; Paz, 1989) to begin developing industrial agriculture, including sugarcane plantations that used large amounts of indigenous forced labor. In the case of the province of Salta, one of the most important sugar mills in the country – “El Tabacal” – was built in the department of Oran at the beginning of the 20th century. It is estimated that it controlled around one million hectares and had around 16,000 workers at harvest time (Weinberg & Mercolli, 2017; Neiburg, 2003) In this context, the sugar mill sought to obtain labor from the indigenous communities of different regions of the Chaco Salteño, the high valleys of Salta and Jujuy as well as Bolivia.

Side by side with the development of these commodity sectors, the Argentinian state encouraged smallholding farmers to aid the “colonization” of the Chaco. Thousands of families emigrated to the Dry Chaco to occupy the territory and begin exploiting land portrayed as “empty” in government propaganda. Today, the descendants of these smallholding colonizers are referred as *criollo*s and their main livelihoods involve small-scale cattle ranching in silvopastoral systems. The relationships between *criollos* and indigenous people are complex and have frequently been tainted by acts of violence and exploitation (Naharro, Alvarez, and Flores Klarik 2010; Klarik 2019). Throughout history, social relations between *criollos* and indigenous communities were marked by disputes over the use of land and natural resources. While *criollo* families tended to settle on specific portions of land and exercised forms of capital accumulation, indigenous communities did not create permanent settlements and pursued diverse livelihood systems, including harvesting natural resources (e.g., wood, fruits), hunting, and fishing (Buliubasich and Rodríguez 2002). The production method of the *criollos* – based mainly on the creation and use of open fields and, in some cases, perimeter enclosure of plots – reduced the space available to indigenous communities for harvesting. This fueled tensions and conflicts over land use that were characterized by persistent unequal power relations between *criollos* and indigenous communities (Naharro, Alvarez, and Flores Klarik 2010). More recently, however, the frictions between *criollos* and local indigenous peoples have diminished considerably. Both groups currently find themselves in a precarious situation regarding land access due to the emergence of a “third-party enemy” – namely, large-scale farms. Conflicts and tensions and collaboration and peer-learning between different indigenous ethnic groups and *criollo* smallholders constitute one of the most salient characteristics of social relationships in the Dry Chaco today. While discriminatory and assistance-oriented practices disadvantage indigenous communities, the arrival of a common enemy who threatens locals’ land access and everyday livelihoods has significantly altered the relationship between indigenous groups and *criollos*. Access to land and official recognition of land tenure are now at the heart of a shared struggle for rights on the part of both groups.

### Legal Response to Deforestation and Land Titling Mechanisms for Indigenous and Peasant Communities

The main legal instrument in Argentina aimed at addressing deforestation on a national level is the National Law N.26.331 (“Ley de Presupuestos Mínimos de Protección Ambiental de los Bosques Nativos”), hereafter referred to as the “Forest Law”. It was adopted in 2007. The Forest Law sets out general principles at the national level and requires individual provinces to pass corresponding implementation regulations at the local level. Overall, the Forest Law builds on two mechanisms: a land-use plan defining conservation priorities (Schmidt, 2015; Alcañiz and Gutierrez 2020) and a fund to promote ecosystem service conservation. The fund is calculated as a proportion of the revenues from commodities sold at the national level. According to federal law, secure land tenure is required to receive payments for ecosystem services (PES) (Núñez-Regueiro et al., 2019); the law in Salta is even stricter, requiring that potential recipients possess an official land title (Aguiar et al., 2018).

Notably, a clear link is made between the Forest Law and the struggle of indigenous and peasant communities for guaranteed access to the land. Under the Forest Law (26.331/2007, art. 5), protection of cultural identity is listed among the forest ecosystem services requiring attention. Further, in the annex of the Forest Law, criteria 10 of forest zonification refers explicitly to the value attributed by indigenous people and peasant communities to the forest in terms of material and cultural reproduction. The Forest Law also mentions the importance of tenure security for these communities in a broad sense. In Salta, law 7543/2008 art. 3 – highlighting the “value and use given to forest by indigenous and peasants communities” – serves as one of the eleven criteria and indicators of environmental sustainability. Chapter 1 of the law on “criteria for zonification” contains additional specifications on forest use by indigenous communities. Nevertheless, there remains a gap between the written support of the forest law for the protection of cultural ecosystem services, on the one hand, and, on the other, the real difficulties indigenous and peasant communities face – most lacking land titles – in accessing PES (Aguiar et al., 2018). Finally, it is worth noting that many NGOs operating in the Chaco Salteño have reformulated their discursive focus to emphasize the importance of the forest for indigenous communities (Castelnuovo Biraben 2020).

### Indigenous and Smallholder Regulations

Like other Latin American countries, Argentina revised its constitution in recent decades to grant legal status to indigenous communities and give them collective rights (Lenton, 2010; Hau & Wilde, 2010). Its national constitution was amended in 1994 to introduce specific protections for indigenous peoples. Article 75/17–18 states that it is the role of Argentina’s Congress:

“*to recognize the ethnic and cultural pre-existence of indigenous peoples of Argentina”* and to “*recognize the legal capacity of their communities, and the community possession and ownership of the lands they traditionally occupy; and to regulate the granting of other lands adequate and sufficient for human development; none of their land shall be sold, transmitted or subject to liens or attachments. To guarantee their participation in issues related to their natural resources and in other interests affecting them”* (*Constitution of Argentina* 1994).

This article provided the legal ground for law 23.302 on indigenous communities. Notably, the constitution partly delegates responsibility for enacting these rights to Argentina’s provinces, as they are considered the owners of the natural resources. As a result, each province has its constitution with a specific chapter on this domain. Under the National Constitution, art. 75 part 17, cited above, Salta’s civil code reformed in 2015 (art 18 of Código Civil y Comercial de la Nación) established the legal category of the common property only for recognized indigenous communities.

At the same time, some observers have criticized the formulation and ambiguity of the corresponding articles contained in Argentina’s constitution. Firstly, for example, the articles’ emphasis on the self-declaration of indigenous communities may allow for the possibility of new communities to emerge. However, it places the responsibility on them to obtain recognition in contexts that are often characterized by significant discrimination and power asymmetries. Secondly, the criteria for land regularization practices are not clearly described, and the civil code does not provide specific instruments to this end – except for law 26.160, an emergency law announced in 2006. The latter’s proposed process for mapping of indigenous land use has not been carried out so far. Thirdly, common property is available only for indigenous communities and not for other groups under the current legal system. As a result, there is a gap between the existence and use of common-pool resources (e.g., pastures, water resources) by diverse groups on the ground and the legal instruments needed to manage this complexity. The case of state land 14 and 55, led by the indigenous organization Lhaka Honat, is an example of this complexity: a provincial decree assigned the land to indigenous and peasant communities, but the process of distributing the land hit an impasse due to lack of procedures to mediate the division of existing common pool resources between two groups. Fourth and finally, the current legal provisions fail to define the minimum parcel of land needed by individuals/groups for adequate, sufficient human development.

One other way for local land users to obtain a land title is through possession: the Argentinean civil code allows for granting of a title to those who can prove that they have used a given parcel of land as the owner for at least 20 years (art. 2351 and 4051 codigo civil). Further, Federal law 26,160 and provincial law 7658, which emerged in response to communities facing eviction, sought to establish mapping processes for indigenous and peasants’ communities, respectively. However, these laws fail to establish a direct path from land (use) mapping to obtaining a land title (Castelnuovo Biraben, 2016; Preci, 2020). Ultimately, Argentina’s executive or judicial authorities always make final decisions on private land/property rights. Today, one of the most important Latin American cases of land users struggling to recognize their rights occurs in the Argentinean Chaco, in the northern part of Salta province (Buliubasich and González 2009; Carrasco 2009; Van Dam 2008). Land in the area is being reallocated between indigenous people and criollos (Castelnuovo Biraben, 2016). Despite Argentina’s existing provincial and national laws aiming to ensure land access to peasant and indigenous communities, land titling remains a complex process requiring time, proactive local leaders, and interventions by external actors such as NGOs or faith-based organizations. While Argentina’s Federal Forest law 26,133 allows local organizations to submit requests for land titling, the lack of resources among said organizations means that without support from NGOs and other catalyzing agents, land titling often remains an unfulfilled promise.

This article explores how local actors’ collective action and coordination and collaboration with external actors can lead to sustainable institutional outcomes. We use the constitutionality framework to highlight different steps in determining local institution-building processes. By focusing on two distinct processes, we show the importance of local intermediaries and identify some weakness in the process – when outside agents have diverging visions and interests. Our research contributes to the debate on participatory development and bottom-up institution building (Mosse et al., 1995; Agarwal, 2001).

Our research also offers a unique case study of collaboration between criollo smallholders and indigenous people. While multiple studies focus on these actor groups individually, literature highlighting the collaboration between them is limited to our knowledge (Hufty, 2008; Seghezzo et al., 2017). The present article seeks to further develop the constitutionality framework by including new elements in the analysis, such as divisions among local actors and patron-client dynamics. These issues are also referred to in the broader literature on the limitations of participatory development (Platteau, 2004; Platteau & Abraham, 2002; Cooke & Kothari, 2001; Mosse et al., 1995).

## Research Methods and Data

This article was prepared in the context of a research project that aims to explore the consequences of deforestation for local actors in the Chaco Salteño, especially the most vulnerable ones. It further examines the role of local actors and institutions in reducing deforestation and environmental degradation in the region. One work package focused on identifying innovative practices in forest management and adoption patterns, including over 12 interviews conducted with local actors – criollo households, indigenous households, and local organizations. Another work package analysed the forest governance policy in Salta province, and it was based on 51 interviews of key policy actors. However, the bulk of the present analysis draws on six semi-structured in-depth interviews conducted by authors with key stakeholders – state agencies, indigenous people and peasant organizations, NGOs, and local politicians – directly involved in recent land-titling and institution-building efforts in Rivadavia Banda Norte and Santa Victoria Este. There were seven respondents, but one did not agree to make the interview recorded; therefore, we could only analyze our notes and not the complete interview responses. We focus on two cases in the Chaco Salteno: one in Santa Victoria Este and the second in the Rivadavia Banda Norte.

Our seven interviewees were asked to answer questions specifically referring to the constitutionality framework. Six of the seven interviews were coded using qualitative content analysis (Schreier, 2012); the resumed coding scheme is presented in Table 1 below, while the complete coding scheme is presented in Appendix A.

**Table 1:** Resumed codebook

	Theme	Definition
**A**	**Emic perception of the need for new institutions**	Reference to the emic need of local actors to access new institutions.
**B**	**Participatory processes of negotiation**	Active participation of local actors in decision making and policy development
**C**	**Preexisting institutions**	Presence of internal rules on the uses of common resources pre-dating statehood.
**D**	**Outside catalyzing agents**	Perceptions and actions of NGOs concerning indigenous and criollo communities
**E**	**Recognition of local knowledge**	Recognition of indigenous and Creole communities’ ways of managing natural resources and having local institutions
**F**	**Higher-level acknowledgment of new institutions**	High-level recognition of new institutions
**G**	**Barriers to the constitutionality process**	Barriers to the constitutionality process such as dependence of provincial policy, divisions among local organizations, capturing and cooptation processes.
**H**	**Approach extension**	Other elements that can influence the constitutionality process are identity, collaborations between indigenous and creole communities, and the absence of the state.

The codebook captures all the dimensions described in the constitutionality framework. In addition, we added several dimensions that we saw as relevant in the context analyzed, seeking to capture possible *barriers* to the process of constitutionality.

The three authors independently coded the six interviews using the codebook shown in Appendix A, Table A1. After the first coding round, the coders met to reconcile any disagreements according to a set procedure. With the supervision of the third coder, coders worked together in pairs of two to solve the following possible cases of disagreement: (a) one coder coded a sentence, and the other did not; (b) two coders coded the same sentence with two different codes. We followed the same procedure for all coding segments to reconcile disagreements between coders. Direct quotes from the stakeholders interviewed were anonymized to ensure the safety of our interviewees. In Appendix A, Table A2 presents a detailed frequency table of all codes used in the six interviews analyzed.

## Results and discussion

This result section is structured as follows. We will discuss the two constitutionality processes from this article with a historical perspective based on primary and secondary literature, interviews conducted, and the author’s own experience in the region. Section 4.3 will compare both cases and highlight what can explain the differences in terms of the six dimensions identified by Haller et al., (2016). Finally, Sect. 4.4 presents the main barriers to constitutionality processes.

### Constitutionality Processes in the Chaco Salteño

#### Constitutionality Case 1: Santa Victoria Este

The first constitutionality process analyzed is in Santa Victoria Este. We focus primarily on the creation of the Mesa de Gestion. It is a body composed of indigenous and *criollo* representatives, which serves as an intermediary in interactions with the state of Argentina and other stakeholders to resolve ongoing land conflicts in the region. We argue that the Mesa de Gestión serves to fill a gap left by the Argentinian state, which withdrew from land titling negotiations against the backdrop of international pressure.

The land conflict in Santa Victoria Este can be traced back to the founding of the organization Lhaka Honhat (“Our Land”) in 1992. The organization was created to obtain a single title of land ownership in the name of all the communities grouped in Santa Victoria Este. Their case for a single title builds on their ancestral use of shared local territory, their use of natural resources for hunting, fishing, and gathering, and a variety of cultural meanings they attribute to the territory (Buliubasich 2013). Together with institutions such as the Anglican Church of Northern Argentina, the Inter-Church Organization for Cooperation and Development (ICCO), Survival International, and Bread for the World, the Lhaka Honhat Association carried out a mapping exercise and a population census. They produced a map illustrating indigenous groups’ communal use of the territory and various forest resources for use in negotiations with the provincial government.

In 2007, following a lengthy negotiation process between the parties involved, the Lhaka Honhat Association and the Organization of Creole Families (OFC) agreed to distribute the state lands they occupied: 400,000 hectares would be allocated to indigenous communities, 243,000 hectares would be allocated to *criollo* families. This agreement between the parties was formalized via Decree No. 2786/07. Afterward, the Government of Salta began a new negotiation stage regarding parcels of land between the communities and *criollo* families, designating the task of the land distribution to the Provincial Executor Unit (UEP). However, implementing this decree has proven challenging due to a lack of financial resources. The UEP’s technical support was required to map criollo families’ land use precisely and help relocate *criollo* families living in indigenous territories. The Mesa emerged in a very complex political and economic context, in which the national government was issuing neo-liberal policies aimed at reducing public spending.

**Fig. 1:**
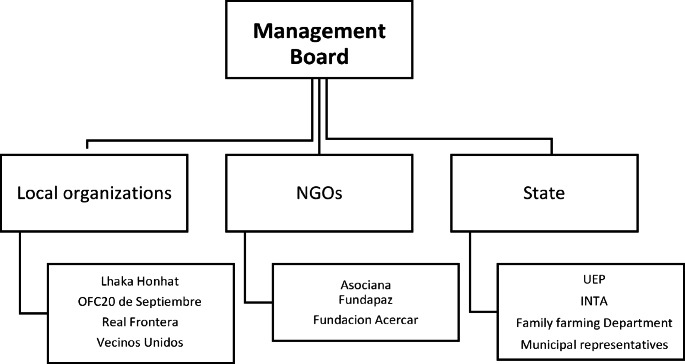
The Mesa de Gestión (based on Saravia 2020)

It was in this context that The “Mesa de Gestión” (hereafter the “Mesa”) was created in 2016 as a heterogeneous space bringing together different stakeholders active in Santa Victoria Este. The main goal of the Mesa was to coordinate the land regularization process. The Mesa is not recognized as an official legal entity. However, it nevertheless serves as a decision and negotiation space that convenes many stakeholders: indigenous and *criollo* organizations, NGOs, and provincial and national state agencies (Fig. 1).

At present, the Mesa serves as a key space for decision-making in the municipality of Santa Victoria Este. It effectively coordinates national and international projects contributing to the resettlement of criollo families and mapping indigenous territories – two essential activities to overcome conflicts in Santa Victoria Este. Since its inception, meetings have been planned to design joint intervention strategies between *criollo* and indigenous representatives. The meetings are held on the third Wednesday of each month. The most recurrent themes are the land regularization process, development projects in the area, possible financing lines, the fight against illegal logging, and interventions by other state bodies that are not part of the Mesa. Decisions in the Mesa are usually taken by consensus and require deliberations among all the participating groups.

Bringing different voices together in one place shifted the power configuration within the municipality, allowing new alliances to be forged between leaders from different organizations (indigenous and criollo). With these new alliances, the Mesa consolidated itself as a working group that debates and actively participates in the land regularization process. In these four years of active functioning, it has managed to establish itself in different spheres of public and political life in Santa Victoria Este, and it is highly representative of all political forces within the municipality. It has become a mandatory step, coordinating activities for all actors involved in the issue of land regularization in Santa Victoria Este.

We think that the following reasons can explain this success:

1. The external pressure from the Inter‑American Court of Human Rights encouraged the development of new deliberations space in the absence of the Argentinian state.
2. Preexisting institutions, in particular for Lhaka Honat an association gathering leaders from five distinct ethnic groups (Boffa 2017).
3. Agreement reached on the solution to the problem of the land-use conflict in Santa Victoria Este, accepted by all actors including *criollo* representatives.
4. Recognition by the actors that the problems that still need to be solved are technical and that a solution can be facilitated through the activities coordinated by the *Mesa*,

#### Constitutionality Case 2: Rivadavia Banda Norte

In this section, we focus on Rivadavia Banda Norte municipality and, more specifically, the efforts by the organizations and NGOs that led to large-scale land titling and the creation of new local organizations. Five parcels of state land (*tierras fiscales*) consisting of 28,323 ha were distributed to *criollos* farmers and indigenous communities during the 1990 and 2000 s. The complete list of land parcels and their relevant areas are listed in Appendix C. In this section, we will describe the historical trajectory of this struggle for land, emphasizing the role of local actors and external organizations that have been acting as catalyzing agents. Several factors might explain the success of the land titling campaign between 1990 and 2010. The political initiative and agenda settings were central to initiating the process. At the same time, the intervention of external actors, such as NGOs was crucial, primarily because of the technical requirements associated with the land tilting process. The third factor of success is the new alliances established at the local level between different groups in response to a common challenge, land eviction due to intense pressure on the land from external investors.

Our interviews identified the following favorable scenario: in the 1990s, the government of Hernan Cornejo (1987–1991) was nearing the end of its term. Governor Cornejo was inclined to make decisions that would benefit the image of his administration. In 1991, a Memorandum of Understanding (MoU) was issued to govern land-related joint work between the state, NGOs, and grassroots organizations. The MoU formally recognized individual land titles for *criollo* families and indigenous communities following their culture and traditional land use. Another essential factor benefitting land titling was that affected communities were settled on a territory free from conflicts with a private owner. As a result, the state had to transfer ownership. It is important to highlight the significance of this event, which emerged at a time when indigenous rights were not yet constitutionally guaranteed.

Second, the vital role of external catalyzing agents: The land titling to indigenous communities and criollo farmers in Rivadavia Banda Norte has required the involvement of a broad range of local actors and several intermediaries (NGOs and faith-based organizations). Argentina’s constitution stipulates that direct involvement of the beneficiaries (indigenous or peasant communities) is required to initiate any land titling process. However, the state recognizes the role of intermediaries in providing technical support for mapping the land claimed. Among such intermediaries, faith-based organizations have played a significant role. In the Chaco Salteño, from the middle of the 20th century onwards, multiple faith-based organizations have been active– predominantly Protestant missionary churches – that forged links with indigenous communities (Cernadas and Lavazza 2017). The work of these missionaries mainly focused on evangelization, taking a paternalistic view of indigenous communities and the “need” to teach them European ways of living (Cernadas and Lavazza 2017). By contrast, local peasant communities – most of them Catholic and practicing mostly sedentary ways of production, whereas Christianized indigenous are predominantly Protestant – were not explicitly targeted by faith-based organizations. An emphasis on securing land titles through various means arose progressively as a priority for faith-based organizations. Interest grew in the territorial titles of the indigenous commissions, a process inaugurated by the Anglican Chaco mission among the Wichí in 1995 (Ceriani Cernadas, 2011).

The third factor absent from the literature on this period is the importance of alliances between indigenous people and *criollo* communities in land titling processes. In Rivadavia Banda Norte, FUNDAPAZ was the first to cooperate closely with the *criollos* in addition to indigenous communities, whereas Tepeyac mainly worked with indigenous groups. In their efforts to obtain land titles, the NGOs sought to identify common solutions for both groups. We argue that indigenous and *criollo* communities’ joint efforts to obtain land titles were very innovative and successful (Fundacion Plurales 2019). However, scholars have overlooked this collaboration based on prior tensions between indigenous people and *criollos* as well as between organizations supporting each group. These under-studied alliances illustrate two critical dimensions of constitutionality: an emic need for new institutions and the importance of preexisting institutions.

The land titling campaigns had long-term local impacts. They led to the creation of new local organizations. For instance, the *coordinadora de tierra*, an umbrella organization created in 2011, regrouped eight *criollo* associations. In 2011, a local activist became mayor of the municipality of Rivadavia Bandar Norte, with the joint support of several indigenous communities and local organizations.

Despite these successes, the land titling procedure in Rivadavia Banda Norte showed some limits: the collaboration between indigenous people and smallholder farmers proved to be efficient, but it was only taking place within a specific geographical and institutional space. For instance, the “Coordinadora de Tierra” emerged as the formalization of the joint efforts between different peasant organizations to fight for land title recognition and the rights of peasant communities. However, the organization was only gathering *criollo* leaders and did not contribute to forging long-lasting alliances with indigenous leaders. The process of constitutionality that resulted in land titling and new leaderships proved to be fragile. Results were achieved when political visions between the municipality and leaders of *criollo* organizations were aligned, but new divisions emerged as soon as political administration changed.

### Cosmparison of Both Processes

Both cases described above qualify as constitutionality processes in that they aim to create new institutions by and for local actors. Both processes were a remarkable result of collaboration between two groups – criollo smallholders and indigenous communities – whose relations were previously antagonistic. Both emerged from emic perceptions of a problem and were built on preexisting institutions. Further, both processes required external catalyzing agents’ intervention that helped make the collaboration possible and sustainable in the long term.

However, as seen in Table 2, our analysis points to differences in the level of development of both processes. The creation of the Mesa appears to represent a completed constitutionality process, as the institution is now established and acknowledged by higher authorities (provincial and/or national institutions). It also fulfills all the conditions by Haller et al., (2016). However, the second case appears to represent a limited or incomplete process of constitutionality.

A hypothesis is that particular political and institutional factors could cause this difference: In Santa Victoria Este, the common indigenous land claims embodied by the organization of Lhaka Honat have been a critical demand of indigenous leaders and activists since 1970. Land claims have structured the municipality’s social and political life for several decades. On the other hand, there are no similar movements in RBN, a municipality where land conflicts are happening locally. This dilution of land conflicts could be reflected in constitutionality, with local leaders that could be less inclined to engage in long-term institution-building in RBN.

Furthermore, conflicting agendas between different intermediaries and the territorialization of their sphere of influence might be a reason for the lack of constant cooperation. The changing vision of NGOs and churches from an evangelical focus to a development focus created tensions between the main organizations operating in Rivadavia Banda Norte and among the Ruta 81 Castelnuovo Biraben, 2016; Castelnuovo, 2013; 2019).

Table 2 summarizes the different factors explaining the successes and limits of the constitutionality process in both contexts. Our analysis indicates that several conditions for “bottom-up institution-building” appear lacking in the case of Rivadavia Banda Norte; the participatory process and the role of outside catalyzing agents have been problematic. In the following sections, we discuss these points further.

**Table 2:** Comparison of processes in both regions

Constitutionality dimensions	Case 1: Santa Victoria Este	Case 2: Rivadavia Banda Norte
**(1) Emic perception of the need for new institutions**	**Yes**. Judicial procedure from Lhaka Honat for recognizing indigenous people’s rights and access to land. Triggered a mirrored demand from criollo families organizations	**Yes**. Collaboration between NGOs and peasant and indigenous communities for recognition of their land rights. The land title was given to both groups (previous fiscal land numbers Nº 15, 16, 17,19, and N 23) Land eviction resistance case: cooperation between indigenous and *criollo* leaders – Caso Dino Salas
**(2) Participatory process addressing power asymmetries**	**Yes**. Negotiation with national- and international-level actors. Strong points: Mesa governs important access to land decisions. Weak points: centralization of the process by the state.	**Limited**. Though there are several positive aspects. *Coordinadora de tierra*, participation of local actors in the legislative process (Ley 7658, Ley 27.118, Mesa de OTBN). Weaknesses: political divisions between different organizations.
**(3) Preexisting institutions upon which to build**	**Yes**. Management rules of *criollo* groups (paraje) and of indigenous communities (to be explored further)	**Yes**. Management rules of *criollo* groups (paraje) and of indigenous communities (to be explored further)
**(4) Outside catalyzing agent**	**Yes**. In Santa Victoria Este, there have been multiple interventions by the central and provincial state, NGOs, inter-American human rights court, churches, etc.	**Limited**. Interventions by several NGOs, churches, and municipal actors. However, we view the plurality of actors and their lack of coordination as a weakness of the constitutionality process (See Sect. 4.3).
**(5) Recognition of local knowledge of resources, creativity, and social learning**	**Yes**. Recognition of the right of indigenous people to have joint ownership of land. Participation of all local actors in provincial and local debates.	**Yes.** Participation in political space (elected as head of the municipality a representative of peasant organization), law 7658 (*criollos* land mapping), participation in provincial forest governance policy forums
**(6) Higher-level recognition of new institutions; support and subsidiarity**	**Yes**. Mesa de Gestión was recognized as an intermediary by different provincial and national authorities, and those stakeholders now regularly participate in meetings.	**Yes.** Several processes are going on, but no extensive geographical coverage. Local-level struggles for control of municipal resources

### Discussion: Critical Elements of Constitutionality

The interviews and our coding methodology enabled us to identify the most salient elements of the two constitutionality processes analyzed and identify weaknesses.

Table 3 summarizes the frequency distribution of coding by typology of organizations. Each theme (code) corresponds to a dimension of the constitutionality framework plus some additional themes that emerged as fundamental in the context analyzed and classified as approach extension.

It shows that most sentences fall under the category “outside catalyzing agents,“ meaning this was a frequent topic in the interviews. Two other frequent topics were participatory negotiation processes and barriers to the constitutionality process.

**Table 3:** Frequency coding bytheme

	Overall frequency	Frequency by NGOs	Frequency by governmental orgs	Frequency by local orgs
Emic perception of the need for new institutions	29	7	10	12
Participatory processes of negotiation	62	14	12	40
Preexisting institutions	13	1	4	8
Outside catalyzing agents	292	97	133	72
Recognition of local knowledge	16	2	0	14
Higher-level acknowledgment of new institutions	51	19	20	12
Barriers to the constitutionality process	91	16	31	44
Approach extension	73	27	25	21
Total	627	183	235	223

#### Role of the catalyzing agents and dependency of local actors

The constitutionality framework emphasizes the perceptions and agency of local actors in the management of common-pool resources (Haller, Acciaioli, and Rist 2016). We applied this framework to analyze two cases of bottom-up institution building in the Chaco Salteño, a vital resource and commodity frontier (Kröger and Nygren 2020). The region is characterized by solid competition over forests between unequal actors (Klarik, 2019; Naharro, Norma, Marcela Amalia Álvarez y Mónica Flores Klarik. 2015) and institutional instability that fuels land tenure insecurity (Valkonen, 2021).

According to Haller et al., (2016), one key element for the success of these processes is the role played by outside actors who act as catalyzing agents, contributing to the establishment of fair platforms and enabling outcomes that otherwise would not occur. This framework closely matches the conditions that we observed in our research context. Here, outside actors aided collaboration in a setting where previously antagonistic indigenous and *criollo* communities developed shared emic perceptions of the need for new institutions to obtain land titles.

Under Argentinian law, indigenous communities and smallholders must initiate a process claim if they want to obtain an official land title. However, these communities typically lack financial means, technical means, and political representation to open the necessary doors. This is especially true concerning state land, where provincial authorities oversee the land titling process. As a result, these communities strongly depend on the outside support from external actors such as NGOs and faith-based organizations. Finally, discrimination against indigenous people and *criollos* and divisions among local organizations also constrain land titling efforts on behalf of these groups.

Our interviews highlight the role of several outside agents in supporting the struggle to obtain land titles for indigenous and *criollo* communities. The first enabling factors were democratization processes and the adoption Argentina’s new constitution in 1994, which recognized indigenous land ownerships and traditional practices.

The second crucial element was the role of NGOs in supporting local groups with organizing, training, and gaining access to the resources necessary to initiate a land title request:

*“A veces pasa que para hacer un trámite tienes que viajar, certificar firmas, ir a un escribano toda la familia, declarar, sacar fotocopias. Y todo eso uno con las ONG puede salvar, haciendo un proyecto” (Interview 1, local activist).*

*“Sometimes you have to travel, certify signatures, go to a notary with your whole family, make photocopies. Through their projects, NGOs can support us with these steps” (Interview 1, local activist).*

Also critical were laws 7658 and 26,160 that reduced evictions and aimed at mapping the land of peasant and indigenous communities (Preci, 2020). However, these laws did not go further than mapping land use, failing to set up an administrative process for land titling on behalf of indigenous and peasant communities (Brent, 2015).

*“No es que fueron y le hicieron un croquis, como la 26.160 hace con las comunidades. Fueron a las casas, le sacaron el punto de GPS para ver donde estaban, se le tomo como declaración jurada lo que registraba. Como cuantos animales tenían, que infraestructura y el promedio de hectáreas que demandaban como suyas”* (Interview 1, local activist).

*“It is not that they went and made a map, as 26.160 does with the communities. They went to the houses, they took the GPS point to see where they were, they took as a sworn statement what they registered. Like how many animals they had, what infrastructure and the average number of hectares they claimed as theirs”* (Interview 1, local activist).

*“Lo que le da fuerza es la ley, el artículo 9 de la ley que evita el desalojo. Pero el relevamiento es como un censo, no te saca ni da el derecho.”* (Interview 3, Government)

*”What gives it force is the law, article 9 of the law that prevents eviction. But the survey is like a census, it doesn’t remove you or give you land rights”* (Interview 3, Government).

It is important to note that NGOs and outside agents were and are aware of their role as intermediaries who enable a fair platform:

*“Nuestro rol no es decirles qué es lo que tienen que hacer sino ayudarles a poner sobre la mesa toda la información posible”* (Interview 6, NGO)

*“Our role is not to tell them what to do but to help them put as much information on the table as possible.* (Interview 6, NGO)

*“entonces nosotros no tomamos partido con nadie. Por supuesto, estábamos más o menos con ellos, ya sé como es la experiencia con criollos e indígenas así que prefiero estar mal con ambos, pero estar con los dos.”* (Interview 5, NGO)

*”So we don’t take sides with anyone. Of course, we were more or less with them, I know what the experience with creoles and indigenous people is like, so I prefer to be bad with both, but to be with both”* (Interview 5, NGO)

At the same time, several interviewees suggested that sometimes NGOs must choose a side and decide to work either with the indigenous communities or with the *criollos.* This can give rise to difficulties. The following section discusses some elements that constrain or block constitutionality processes.

### Barriers to the Constitutionality Process

Our analysis highlighted several critical weaknesses of the constitutionality process in the region. These were, first, lack of deliberative spaces overseen by neutral agents; second, the judicialization of land title access; third, patron-client dynamics between local organizations and local politicians.

#### Capture of the Constitutionality Process by Outside Agents

Our interviews suggest that things work smoothly when the various catalyzing agents share the same visions and interests, but the constitutionality process grinds to a halt when they are divided. The case study in Rivadavia Banda Norte indicates that a strong alliance between NGOs, *criollos*, and indigenous people created the conditions to pressure the provincial state to allocate a large amount of public land in a short time. However, land allocation slowed down considerably as soon as tensions arose between the NGOs involved. Robust and unified advocacy and lobbying towards the provincial government were crucial to the process’s success. The situation remains fragile because it depends on the capacity to achieve consensus in a territory where the different state and civil society organizations are fractured (Castelnuovo 2019).

Finally, patron-client dynamics were highlighted by interviewees as another very problematic issue, echoing critical participatory development elsewhere. In particular, many respondents highlighted how local representatives tend to cater to and divide different constituents – e.g., highly vulnerable indigenous or peasant communities – before and during election cycles. The isolated benefits that local politicians offer to different groups ultimately undermine broader collective action and collective bargaining opportunities.

#### Lack of Deliberative Space

In Rivadavia Banda Norte, although the state has been central to transferring land titles to local smallholders and indigenous communities, it often intervened only after an agreement is reached between the different parties involved (*criollos* and indigenous communities). Hence, the state only provided marginal support in the land regularization process. In Santa Victoria Este, the lack of a mediator and/or space for discussion was initially even more problematic. However, it eventually gave rise to a constitutionality process in the form of the Mesa de Gestion. Indeed, the Mesa was created because it *filled a gap*, giving local actors a space to meet to discuss ongoing conflicts. It was possible to create this type of institution in Santa Victoria Este, a municipality in which land conflicts overlap with the municipality boundaries because of the shared land claim of Lhaka Honat. Creating such an institution in Rivadavia Banda Norte is far more complicated due to the high number of small-scale land conflicts and the division of the territory between public and private land.

Overall, the persistence of many land conflicts in the region can be explained, at least in part, by the absence of a fair platform for deliberation and the irregularity of support from provincial authorities (Chappuis 2021). The lack of state-led negotiations and action on regularization has caused a proliferation of conflicting cases between indigenous communities and *criollo* families. Our interviews highlighted the critical role played by the provincial government in granting access to land titles – whether through official or unofficial channels. For example, the interpretation of state and provincial actors prioritizes the resolution of specific land conflicts over others. In this way, the state’s ability to “tip the balance “and facilitate the negotiation process should not be underestimated. To date, however, local authorities’ actions and prioritization of conflicts have not resulted from transparent deliberations but rather obey the logic of political interests. This further marginalizes local actors who lack the political support needed to resolve their land claims.

#### Judicialization of the Process of Land Title Access

As discussed above, the Argentinean state is generally willing to engage in the transfer and distribution of public land to *criollo* and indigenous communities if catalyzing agents properly support local actors. However, our interviews also indicated that provincial authorities often fail to intervene in conflicts that eventually arise between *criollo* or indigenous communities and private companies or large-scale landowners. Instead, the state tends to redirect conflicts to the judicial system in a process referred to as the “judicialization of land conflicts” by Mioni et al., (2013). Although indigenous people and criollos are protected in principle by several laws, the resulting judicialization is characterized in practice by power and resource asymmetries that often result in the court’s ruling in favor of large-scale landowners. This process leaves little room for local actors to create coalitions and build institutions to serve their interests.

Lastly, the judicialization of land claims may also contribute to further marginalizing the ontologies of disadvantaged groups like indigenous communities and *criollo* smallholders. In line with Gambon (Gambon and Rist 2019; 2018) and Viveiros de Castro (de Castro 1998), we argue that the judicialization of land conflicts serves to crystallize “communicative disjuncture” between different stakeholders who subscribe to radically different ontologies. In this way, judicialization significantly hinders the constitutionality process, as it strongly undermines fair participatory approaches in settings characterized by power asymmetries and ontological differences between distinct groups (Gambon, 2020).

## Conclusions

This paper used the constitutionality framework to analyze collective action and institutional development in two separate cases in the Chaco Salteño. We discussed how constitutionality could be understood as a process occurring at different levels of implementation, depending on specific local conditions and external factors that can either facilitate or impede the process. We argued that the bottom-up institution-building has almost been completed in one case, that of Santa Victoria Este, but has been halted in another case, Rivadavia Banda Norte. By comparing two case studies from the same region, our analysis highlighted similarities and differences between the two processes. We focused our analysis and discussion on the most critical elements that facilitated or hindered both processes by using results from a strict interview coding procedure on participants and experts who were active in both processes.

We showed that the intervention of external agents and catalyzing factors could play an essential role in leading to a constitutionality process. However, the case of land titling in Rivadavia Banda Norte showed the limits of the land titling process, albeit advances in constitutionality. However, it also suggests that continuous involvement of external agents in such contexts might be needed to maintain a platform over time. The Mesa de Gestion showed that the issue of boundaries is also crucial. In RBN, land conflicts exist in multiple areas, weakening the constitutionality process. In the case of Santa Victoria Este, the boundaries of land conflict are juxtaposed with concrete political and social entities.

We showed that despite their history of conflict, *criollos* and indigenous communities could and do collaborate, yielding actual results in terms of institution-building. However, there are weaknesses in our analysis. Firstly, we considered *criollos* and indigenous communities as relatively consistent, unified categories or “blocs” when in reality, these groups feature significant inner differentiation based on shifting alliances and power relations. In addition, both groups are dynamic and characterized by unique struggles to recognize their cultural identities – processes that may have important ties with constitutionality and institution-building that require further exploration.

## Data Availability

Data available on request from the authors. The data that support the findings of this study are not openly available to protect the confidentiality and personal information of respondents. Data derived from the study and analyzed are available from the corresponding author upon reasonable request.

## Appendices

### Appendix A: Codebook

**Table:**

	Theme	Definition	Example
**A**	**Emic perception of the need for new institutions**		
A1	Request for land title	Referencia a la necesidad emica de los actores locales a tener acceso a un titulo propiedad	la necesidad surgió de las organizaciones cuando fuimos (3)
A2	Local actors in the decision-making spaces	Participación de los actores locales en los procesos de decisiones políticas	no tienen que tener intermediarios, tienen que estar presentes ellos tomando y respetan la decisión. (I4 p.3)
**B**	**Participatory processes of negotiation**		
B1	Participation	Participación activa de los actores locales en las tomas de decisiones y elaboraciones de políticas publicas	no tienen que tener intermediarios, tienen que estar presentes ellos tomando y respetan la decisión. (I4 p.3)
B2	Collective organizations of small-scale cattle ranchers	Rol protagónico en las actividades desarrolladas por las organizaciones campesinas criollas	el censo ya se hizo acá. Lo hicieron las organizaciones, incluso el formulario. En ese proceso participamos mucho. (I1)
B3	Collective organization of indigenous community	Papel de las organizaciones indígenas en la promoción de derechos en el reconocimiento de titulación de tierras o otros	as comunidades estaban básicamente organizadas a través de la Iglesia Anglicana. (Interview6)
**C**	**Preexisting institutions**		
C1	Rules on the use of common resources	Presencia de reglas internas en los usos de recursos comunes prexistentes a la conformación del estado	hay lugares que si o si van a ser de uso comunitarios como las aguadas, porque acá no hay agua. (I1, p4)
C2	Collective choice arrangements	Reglas internas que facilitan el uso de los recursos entre los usuarios del monte	Algunos criollos ya tienen los acuerdos implícitos, incluso nosotros antes del relevamiento, para lado del Bermejo (I1, p5)
C3	Barriers to the constitutionality process	Referencias a procesos o dinámicas que no facilitan el despegue de procesos de constitutionality	Algunos dirigentes no tienen la madures suficiente para saber aceptar que se puede trabajar sin la necesidad de ser amigos
**D**	**Outside catalyzing agents**		
D1	Role of large producers	Acciones de los grandes productores agrícolas sobre las comunidades indígenas y criollas	por la influencia de la gente que está viniendo de otro lado, cierra sus parcelas. (I2)
D2	Role of NGOs	Percepciones y acciones de las ONGs en relación a las comunidades indígenas y criollas	[NGO T] apoyo técnico productivo pero solo con comunidades (I4)
D3	International community pressure	Referencia al papel y presencia de organizaciones internacionales en relación a las comunidades indígenas y criollas	a cooperación le daban pena, veían más pobres a los indígenas. (I4, p3)
D4	Role of Government Nacional	Papel del gobierno Nacional en relación a las comunidades indígenas y criollas en particular en el proceso de entrega de tierra y de reconocimiento de los derechos.	El actor que hoy empieza a ser fuerte, bueno, siempre fue fuerte el comerciante, el intermediario. Bueno, el Estado con sus diferentes formas. (I6)
D5	Role of Government Provincial	Papel del gobierno provincial en particular en el proceso de entrega de tierra y de reconocimiento de los derechos de las comunidades indígenas y criollas.	porque es el gobierno el que iba a otorgar los títulos. (I5)
D6	Law 26.160	Aspectos positivos o negativos de la ley 26.160 en el proceso de entrega de tierra y de reconocimiento de los derechos de las comunidades indígenas.	26.160, ayuda a dar información para ver quiénes son las partes que tienen que negociar (I4)
D7	Law 7658	Descripción y/o valoración de la ley 7658 en aspectos positivos o negativos en referencia al en el proceso de entrega de tierra y de reconocimiento de los derechos.	Inclusive Antolín de los Blancos, él tiene orden de desalojo pero no se ejecuta porque está en proceso la 7658, el artículo 7 que es la previsión de desalojo. (I1)
D8	Law 27,118	Descripción y/o valoración de la ley 27,118 en aspectos positivos o negativos en particular en reconocimiento de los derechos de los derechos de las comunidades indígenas y criollas.	nosotros (las organizaciones de Morillo) estamos con el foro de la agricultura familiar, que es a nivel provincial y nacional. (I1 p15)
D9	Ley 26.331	Descripción y/o valoración de la ley 26,331 sea en aspectos positivos o negativos en particular en reconocimiento de los derechos de los derechos de las comunidades indígenas y criollas.	Pero si ley de bosque, bosque y comunidad, todo tipo de proyecto que involucra a los bosques, los que se manejan vía medio ambiente exigen un paso por la oficina legal. (I5 p12)
D10	Art. 75 Argentina Constitution	Descripción y/o valoración de la reforma de la constitución argentina en el reconocimiento de los derechos de los derechos de las comunidades indígenas	el año 1994 se modifica la Constitución Nacional que se incorpora la propiedad aborigen (I5)
D11	Role of faith-based organizations	Descripción y/o valoración de la de las iglesias y sus organizaciones afiliadas en relación a las comunidades indígenas y criollas	(Monjas) ellas querían que se trabaje sobre la entrega de tierras fiscales (I6)
D12	Casos Judicializados	Descripción de la importancia de la presencia del papel judicial en el proceso de acceso al título de tierras el reconocimiento de los derechos de los derechos de las comunidades indígenas y criollas.	Lleva como 5 o 6 años en el juicio y va perdiendo dos veces ya.(I2, p6)
**E**	**Recognition of local knowledge**		
E1	Recognition of resources management	Reconocimiento de los modos de uso de las comunidades indígenas y criollas de los recursos naturales	No tiene infraestructura, lo único que puede tener es su casita, un pozo de agua y tal vez 2 o 3 potreros, pero no llega a cubrir (I1, p.2)
E2	Different ontologies	Presencia de diferentes ontologías entre las comunidades indígenas y criollas	se nota desproporcionado, porque las comunidades al ser comunitarias, toda la comunidad puede tener 300 familias. En cambio, vemos pocos criollos, son 6… porque están dispersos ellos. (I2, p6)
**F**	**Higher-level acknowledgment of new institutions**		
F1	International Court case de LH N° 12,094	Descripción y/o valoración del caso internacional de Lhaka Honat contra argentina	la denuncia que se hace ante la Corte Iberoamericana afecta al gobierno nacional, y éste le exige al gobierno provincial que dé una solución porque las tierras son de la provincia de Salta. (I4, p5)
F2	Local governance board (Mesa de Gestión Santa Victoria Este)	Descripción y/o valoración del papel de la Mesa de gestión de Santa Victoria Este	la verdad sé que hay una mesa de gestión pero no sé cómo funciona. Me parece que está muy lindo pero queda solo en reuniones y no se concreta. (I5,p2).
F3	Court Case Dino Salas and Cata Riera (RBN, Ruta 81)	Descripción y/o valoración del caso Dino Salas y su impacto sobre los derechos y el proceso de acceso al título de tierra	mi compañero Riera, que salió en todos los medios, querían sacarlo a la fuerza con policías, con infantería, era como una guerra. (I2)
F4	Local governance board (Coordinadora de tierra Morillo)	Descripción y/o valoración de la Coordinadora de tierra Morillo y su impacto sobre los derechos y el proceso de acceso al título de tierra	
F5	Local governance board (Mesa de agua Morillo)	Descripción y/o valoración de la Mesa de agua Morillo y su impacto sobre los derechos y el proceso de acceso al título de tierra	Es un espacio donde tanto indígenas como criollos discuten cómo se distribuyen las obras. (I6)
F6	Land title to indigenous and criollo community from fiscal land (Ruta 81)	Descripción y/o valoración del proceso de acceso al título de tierra en los fiscales presentes en la ruta 81	pero todos están entregados con títulos, todos: 15, 16, 17, 21, 22 y 23 (I6)
F7	Nestedness	Referencia al principio de Ostrom sobre common pool resources management	
**G**	**Barriers to the constitutionality process**		
G1	Patron–client dynamics	Presencia de dinámicas clientelares	Hay mucho clientelismo político, hay como una trampa muy difícil de romper. (I6)
G2	Dependency on provincial politics	Dependencia de la política provincial sobre los derechos y el proceso de acceso al título de tierra de las comunidades indígenas y criollas	Con el Estado es así, los que se juegan más la elección es en donde hay conflictos, los diputados, senadores, hay más parte…. desde la gente hay mucho “sí, sí, pero no sos mi amigo en el fondo”. (I5)
G3	Division among local actors	Divisiones entre las organizaciones locales (comunidades indígenas y criollas)	si los poseedores de los fiscales no están bien divididos, no están de acuerdo, no tienen su proporción de tierra, hay conflicto. (I1)
G4	NGOs capturing local institutions	Referencias a la presencia de ONGs que capturaron los procesos de constitutionality	
**H**	**Approach extension**		
H1	Identity	Proceso de identidad de las comunidades indígenas y criollas ( procesos de constitutionality)	Por las luchas, ya hay un concepto, un imaginario acerca de que si vienen de otra manera van a tener problemas; esta el imaginario de la gente que sabe. (I 2)
H2	Collaboration between indigenous people and criollos	Colaboraciones entre las comunidades indígenas y criollas para los procesos de constitutionality.	Lakha Honat, en Santa Victoria empezó a avanzar en el 2001, van a decir eso si son justos, cuando criollos e indígenas empiezan a trabajar juntos para presionar al gobierno. (I5)
H3	Ausencia del Estado	Referencia a la ausencia del estado en procesos relacionados a los derechos y el processo de acesso al título de tierra de comunidades indigenas y criollas	hace décadas que la provincia no se ocupaba de sus tierras (I4)
H4	Fair platform	Presencia de una espacio de negociación donde los distintos actores se pueden confrontar de forma equitativa	Con [local activist 2]se crea un consejo asesor, se intenta invitar a los empresarios aunque no venían; pero incluso con ese actor, volviendo al Estado, yo creo que con el cargo municipal es complicado, complejo, no hay una buena relación, no hay respeto, no hay amistad, sino es solo alguien a quien se le puede sacar cosas y se hace reclamos. (I5)
H5	Otros	Frases que son importantes pero presentes en los códigos arriba enumerados.	ero hasta ahora escucho que la gente, cuando le preguntan su ocupación, dicen que no tienen; la actividad del campo no la ven como económica.(I2)

### Appendix B: Frequency Table Distribution

**Table:**

	Theme	01.gov.	01.ngo	01.local.org	02.local.org	02.ngo	02.gov	
A	Emic perception of the need of new institutions							
A1	Request for land title	3	1	2	3	2	3	14
A2	Local actors in the decision-making spaces	2	3	4	3	1	2	15
	Total	5	4	6	6	3	5	29
B	Participatory processes of negotiation							
B1	Participation	0	0	0	0	4	0	4
B2	Collective organizations of small-scale cattle ranchers	4	1	32	7	3	2	49
B3	Collective organization of indigenous community	3	2	0	1	0	3	9
	Total	7	3	32	8	7	5	62
C	Preexisting institutions							
C1	Rules on the use of common resources (aclarar)	0	0	3	0	1	2	6
C2	Collective choice arrangements	0	0	2	0	0	1	3
C3	Barriers to the constitutionality process	0	0	1	2	0	1	4
	Total	0	0	6	2	1	4	13
D	Outside catalyzing agents							
D1	Role of large producers	1	0	11	2	0	5	19
D2	Role of NGOs	1	22	5	2	2	24	56
D3	International community pressure	0	1	0	0	0	1	2
D4	Role of Government Nacional	1	6	6	0	0	8	21
D5	Role of Government Provincial	12	3	15	0	13	17	60
D6	Law 26.160		0	5	0	1	0	6
D7	Law 7658	40	0	5	1	5	0	51
D8	Law 27,118	0	0	1	0	0	0	1
D9	Ley 26.331	3	10	1	1	9	1	25
D10	Art. 75 Argentina Constitution		2	2	0	2	1	7
D11	Role of faith-based organizations	2	10	5	0	1	12	30
D12	Casos Judicializados	4	0	6	4	0	0	14
	Total	64	54	62	10	33	69	292
E	Recognition of local knowledge							
E1	Recognition of resource management	0	0	4	0	0	0	4
E2	Different ontologies	0	2	4	6	0	0	12
	Total	0	2	8	6	0	0	16
F	Higher-level acknowledgment of new institutions							0
F1	International Court case de LH N° 12,094	0	0	0	0	2	2	4
F2	Local governance board (Mesa de Gestión Santa Victoria Este)	2	0	0	0	0	1	3
F3	Court Case Dino Salas and Cata Riera (RBN, Ruta 81)	0	0	2	1	0	0	3
F4	Local governance board (Coordinadora de tierra Morillo)	0	0	0	0	0	0	0
F5	Local governance board (Mesa de agua Morillo)	0	0	0	0	8	0	8
F6	Land title to indigenous and criollo community from fiscal land (Ruta 81)	4	9	3	6	0	11	33
F7	Nestedness	0	0	0	0	0	0	0
	Total	6	9	5	7	10	14	51
G	Barriers to the constitutionality process							0
G1	Patron–client dynamics	0	7	3	3	0	4	17
G2	Dependency on provincial politics	0	3	7	0	1	12	23
G3	Division among local actors	0	4	23	4	1	13	45
G4	NGOs capturing local institutions	0	0	0	4	0	2	6
	Total	0	14	33	11	2	31	91
H	Approach extension							0
H1	Identity	0	1	6	7	0	0	14
H2	Collaboration between indigenous people and criollos	2	6	1	2	6	12	29
H3	Ausencia del Estado	0	5	0	0	3	0	8
H4	Fair platform	0	0	0	0	5	6	11
H5	Otros	0	1	4	1	0	5	11
	Total	2	13	11	10	14	23	73
Overall total		**84**	**99**	**163**	**60**	**70**	**151**	627

### Appendix C: Tierras fiscals distributed

**The constitutionality process analyzed in Rivadavia Banda Norte includes transferring the following provincial land property to indigenous and criollos organized in associations.**.

Fiscal No. 15 located in Capitán Pagé is made up of 23 individual number plates. The Lewetes Kayip Neighborhood Centre community is located within it, where the provincial state authorities have recognized 9,393 hectares.

Fiscal No16 is made up of five individual registration numbers. One of them still appears in the name of the Government of the Province of Salta.

Fiscal No 17, located in Coronel Solá, is made up of 25 individual registration plates. The community of Centro Vecinal Wichi Lewetes San Patricio, with 3,729 recognized hectares.

Fiscal No 19 located in Coronel Sola is made up of 16 individual number plates. The community Centro Vecinal Wichi Lewetes is located here, with 4,379 recognized hectares.

Fiscal Noª20 conformed by five individual Families. The community Wichi Lahco Pluma de Pato is located here, with 3,000 recognized hectares.

Fiscal No 22 is made up of 28 individual registrations. In the first of community, 3,878 hectares were recognized. No information is available for the second. The Wichi Lewetes Aboriginal Community “La Cortada” and the Wichi Lewetes Letsenwat Aboriginal Community “Pozo El Chañar” are located here.

Fiscal No 23 is made up of 21 individual registration numbers. The community Centro Vecinal Wichi Lewetes Kalehi is located in the same area, with 3,944 recognized hectares.

## Footnotes

1 The 2002 Agrarian Census indicates that there are 132 units labelled Limited liability companies (*Sociedades de responsabilidad limitada*) or Public Limited Company (*sociedades anónimas y en comandita por acciones*), with average area of 12,900 hectares, Recent literature as well as project such as the Land matrix indicate that the number of companies has increased significantly since then (https://landmatrix.org/, https://datos.gob.ar/dataset/agroindustria-censo---explotaciones-agropecuarias-cna-02 ).
